# Oral health promotion: the economic benefits to the NHS of increased use of sugarfree gum in the UK

**DOI:** 10.1038/sj.bdj.2016.94

**Published:** 2016-02-12

**Authors:** L. Claxton, M. Taylor, E. Kay

**Affiliations:** 1 York Health Economics Consortium, Enterprise House, Innovation Way, University of York, Heslington, York, YO10 5NQ; 2 Plymouth University, Peninsula Dental School, John Bull Building, Tamar Science Park, Research Way, Plymouth, Devon, PL6 8BU

## Abstract

**Introduction** The effect of sugarfree gum (SFG) on the prevention of dental caries has been established for some time. With increased constraints placed on healthcare budgets, the importance of economic considerations in decision-making about oral health interventions has increased. The aim of this study was to demonstrate the potential cost savings in dental care associated with increased levels of SFG usage.

**Methods** The analysis examined the amount of money which would hypothetically be saved if the UK 12-year-old population chewed more SFG. The number of sticks chewed per year and the caries risk reduction were modelled to create a dose response curve. The costs of tooth restoration, tooth extraction in primary care settings and under general anaesthetic were considered, and the effects of caries reduction on these costs calculated.

**Results** If all members of the UK 12-year-old population chewed SFG frequently (twice a day), the potential cost savings for the cohort over the course of one year were estimated to range from £1.2 to £3.3 million and if they chewed three times a day, £8.2 million could be saved each year. Sensitivity analyses of the key parameters demonstrated that cost savings would still be likely to be observed even in scenarios with less significant increases in SFG use.

**Conclusion** This study shows that if levels of SFG usage in the teenage population in the UK could be increased, substantial cost savings might be achieved.

## Introduction

The effect of sugarfree gum (SFG) on the development of dental caries has been widely studied. Evidence indicates that chewing SFG, particularly after consumption of food, can reduce the development of dental caries.^1^,^2^ Both the incidence and rate of progression of dental decay are reduced through the mechanism of increased salivary flow. Saliva neutralises plaque acids, remineralises tooth enamel and helps to remove food debris from the mouth and teeth. The more stimulated saliva is produced, the more pronounced these effects. Saliva is, therefore, essential for caries prophylaxis,^3^,^4^ and so by stimulating the production of saliva, SFG reduces the incidence of caries.^5^

A number of studies have investigated the relationship between chewing SFG and reduced levels of caries, and reviews have established that there is a causal relationship between the chewing of SFG and plaque acid neutralisation, reduction of tooth demineralisation, maintenance of tooth mineralisation and reduction of oral dryness.^6^

The NHS spends £3.4 billion per year on primary and secondary care dental services for adults and children in England, with over one million patient contacts with NHS dental services in England each week.^7^ Dental care services, like other parts of the UK NHS, face challenges in closing the projected 2021/22 funding gap of £30 billion. The financial impact of poor dental health to patients can also be substantial, and has been demonstrated to be increasing year on year. In 2013, patient charge revenue increased overall by more than £27 million to £685 million.^8^ The economic burden of dental diseases is by no means limited to the UK: it was recently estimated that direct treatment costs due to dental diseases worldwide were US$298 billion per year; approximately 4.6% of global health expenditure. Indirect costs relating to productivity losses due to absenteeism from school and work were also substantial, and amounted to US$144 billion per year.^9^

With increased constraints on healthcare budgets, the importance of economic considerations in decision-making about new and existing health interventions has increased. One method of determining the value for money of an intervention is to develop an economic model to predict the improved health outcomes and consequent reduced health care costs that are likely to be associated with the intervention. Models are a useful tool for representing the detailed and complex 'real world' with a more simple and understandable structure. While they do not create an exact replica of the real world, they are useful for demonstrating the relationships and interactions between various different factors.

Economic analyses of the cost-effectiveness of interventions preventing oral disease are rare. To our knowledge, none have been conducted that investigate the economic impact of the use of SFG. The purpose of this research was to estimate the potential cost-savings to the NHS that could be realised through increased use of SFG among the 12-year-old population in the UK due to the resultant reduction in tooth decay and subsequent dental procedures.

## Methods

The majority of evidence about the impact of oral health interventions has been derived from child and teenage populations. This is largely due to the fact that the majority of tooth decay is considered to develop before the age of 15. In addition, teenagers are the demographic group most likely to use SFG and detailed data regarding 12-year-olds' dental health is available. Therefore, the analysis focused on the reduction in dental care needs that could potentially be observed in the population of 12-year-olds in the UK, if more SFG were chewed.

This economic model was developed to estimate the reduction in expenditure on dental treatment that would occur if SFG were chewed more frequently by the 12-year-old population. The model compared the dental health of 12-year-olds currently, to various hypothetical situations with higher levels of SFG use, and estimated the total annual expenditure on the treatment of dental caries which could be avoided by increasing SFG usage. Outcomes were assessed over a one-year time horizon. The perspective taken was that of the NHS – costs to the consumer for the purchase of SFG or to the body bearing the costs of a promotional campaign to increase SFG usage were not included. Patient charges for dental treatment were also excluded.

The primary analysis assumed full compliance and uptake of the SFG regimen within each scenario (that is, in a scenario looking at the benefit of SFG used twice a day, it was assumed that all individuals use SFG twice a day for the full duration of the model timeframe).

Current SFG usage in the UK was estimated using a consumer survey of a nationally representative sample of people aged 10 to 59 years. The model then compared the observed situation among teenagers, to a hypothetical population of teenagers with substantially greater use of SFG (Table 1). The budget impacts of various degrees of increase in chewing frequency were considered.

Baseline risk of disease was taken from the Dental Public Health Epidemiology Programme.^10^ The dataset used was the population of 12-year-olds in England, in 2009. This dataset was also used to determine the proportion of children with tooth decay who receive treatment, and the type of treatment that they receive (Table 2).

A rapid literature review was undertaken to identify clinical studies reporting the impact of chewing SFG on the development of caries, compared with non-chewing controls. A pragmatic electronic search of PubMed was undertaken to identify any relevant studies. A total of 15 studies were reviewed in full.^11^,^12^,^13^,^14^,^15^,^16^,^17^,^18^,^19^,^20^,^21^,^22^,^23^,^24^,^25^ Characteristics of the included studies are presented in Table 3.

No UK-specific studies were identified in the review. Studies considered for use within the model were those published since 2000, resulting in a number of the listed studies being excluded from consideration for inclusion in the analysis.^11^,^12^,^13^,^14^,^15^,^16^,^17^,^18^ Those studies identified in the review that evaluated outcomes for deciduous teeth were also excluded.^22^,^23^,^25^ One study did not include a 'no gum' group, and was excluded for consideration on those grounds.^24^ After these studies had been excluded from consideration, three studies from different European settings, including Hungary, Lithuania and Estonia, were evaluated.^19^,^20^,^21^ The baseline levels of decay varied across each of the studies, and different criteria were used to identify decay, with some including incipient caries and some not. The risk reduction in caries varied from 54% (Alanen *et al*., 2000^19^) to 25% (Machiulskiene *et al*., 2001^21^). The three-year study by Machiulskiene *et al*., 2001^21^ of school children based in Lithuania aged 9 to 14, was considered to be the most appropriate for estimating the impact of SFG in the model population. In the study, the mean decayed, missing, filled tooth surfaces (DMFS) at baseline was 14.3 (SD 8.0) for the 'no gum' group and 13.2 (SD 8.9) for the 'gum chewing' group. This study showed the lowest risk reduction of the three relevant studies, it was therefore selected so that the model examined the most conservative estimate of the potential cost savings. Given the differences between the caries susceptibility in Lithuania and the UK, and the relationships between baseline risk and risk reduction, the risk reduction parameter was explored in a sensitivity analysis.

A number of studies have demonstrated the existence of a dose response relationship, that is, the more gum was chewed, the lower the rates of decay. In the study by Tao *et al*., 2013,^25^ it was shown that daily consumption of two and three pieces of gum led to 33% and 58% reductions in the DMFS increment, respectively. The caries benefit estimated by Szöke *et al*., 2001 was based on a chewing duration of twenty minutes, while another study showed similar significant benefits from chewing for only five minutes.^15^ It was therefore assumed that duration of chewing had little impact on the amount of risk reduction of dental caries.

**Figure 1:**
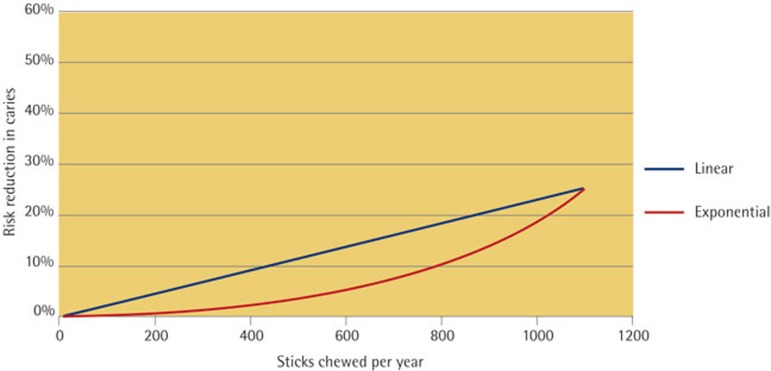
Frequency-response relationship

Since evidence on the benefits of chewing SFG less than three times per day was not available in the Machiulskiene study,^21^ two hypothetical scenarios were developed in order for the model to take account of uncertainties about the exact nature of the dose-response of caries to chewing frequency. The models were a linear frequency response and an exponential frequency response; both of which were considered to be plausible representations of the dose response relationship between caries risk reduction and frequency of use of SFG (Fig. 1).

The costs associated with the treatment of tooth decay that were incorporated into the economic model included the costs to the NHS of tooth restoration and tooth extraction. The cost of a restoration or an extraction in the primary care setting was estimated to be £75, and it was assumed that 20% of extractions take place under local anaesthetic in that setting. The remainder of tooth extractions were assumed to take place under general anaesthetic. Inpatient treatment for a dental extraction was estimated to cost around £1,165.^26^ Of those patients with caries experience, the proportion with restorations was used to estimate the average spending on restorations per case of caries, and the proportion with extraction experience was used to estimate the average spending on extractions per case of caries. All of these estimated parameters were subjected to a sensitivity analysis.

## Results

The results were based on the analysis of the population of 12-year-olds in the UK. The size of this population is 684,817.^27^ The results are presented for both the linear and the exponential frequency response models. The model estimated that the current total expenditure in this population on extractions and restorations is £33.4 million (£33,375,698) per year.

The analysis indicated that if all members of the 12-year-old population chewed SFG frequently (twice a day) the subsequent prevention of dental caries could save between £1.2 and £3.3 million per year and if they chewed SFG after every meal (three times a day), that over £8 million could be saved each year.

Results are presented for three potential scenarios – the first corresponding to frequent SFG use by everyone in the model population, that is, an increase in chewing frequency to all members of that population using SFG twice per day (Table 4a). The second scenario examined the impact of an increase in consumption across the model population of one additional use per day (Table 4b). For instance, a current non-user would chew one piece of SFG per day, whilst a frequent user, currently chewing twice a day, would now chew SFG three times a day. The third scenario modelled the impact of increasing SFG use across the model population to three times per day (Table 4c).

The cost savings were less when the exponential rather than the linear model were applied, but were still considerable. Thus, depending on the degree to which SFG use increased, and the dose response model applied, cost savings ranged from £1 million per year (exponential model and one additional SFG use) to £8.2 million per year (exponential frequency model plus three times per day SFG use).

### Sensitivity analysis

Sensitivity analyses were conducted to explore the impact of the uncertainty within the model resulting from assumptions it was necessary to make in order to construct it. Sensitivity analysis involves varying a value in the model by a given amount, and assessing the impact that the change has on the results.

A number of parameters were considered to be associated with a considerable level of uncertainty, including the relative risk of decay associated with SFG, the cost of a restoration, the proportion of decayed teeth that receive restorative treatment, and the proportion of the population who modify their behaviour. These sensitivity analyses were applied to the results for a population of 12-year-olds using SFG twice each day.

Analysis was also undertaken to assess the potential cost savings if SFG had a lower caries protective effect than estimated from current evidence. The analysis demonstrated that cost savings could still be observed even when benefit of SFG was minimal. The potential cost savings were estimated to be between £0.1 and £0.2 million per year when the caries risk reduction resultant from SFG use was assumed to be only 1%, and between £0.7 and £1.3 million per year when the risk reduction was assumed to be only 10%. In reality, it has been confirmed by our review of literature that the risk reduction is likely to be considerably higher than this.

There are no nationally published figures for the actual cost of each dental procedure. Therefore, there exists some uncertainty in the most appropriate value to use in the economic analysis. The sensitivity analysis explored what happens to the total cost saving when the cost of treatment changes. The total cost saving increases as the cost of a tooth restoration increases. The potential cost savings were estimated to be between £0.9 and £2.6 million per year when a procedure is assumed to cost £30, and up to £1.4 and £3.9 million per year if a procedure costs £120.

In the base case analysis, evidence from a nationally-representative dataset was used to estimate the proportion of 12-year-olds with tooth decay who had an extraction or a restoration, and in the first analysis 60% of decayed teeth were assumed to be restored. Total cost savings were demonstrated to increase as the frequency of restoration of decayed teeth increases. The potential total cost savings were estimated to be between £0.9 and £2.4 million per year when only 10% of decayed teeth were restored, and between £1.4 and £3.9 million per year if 100% of decayed teeth were restored.

**Figure 2:**
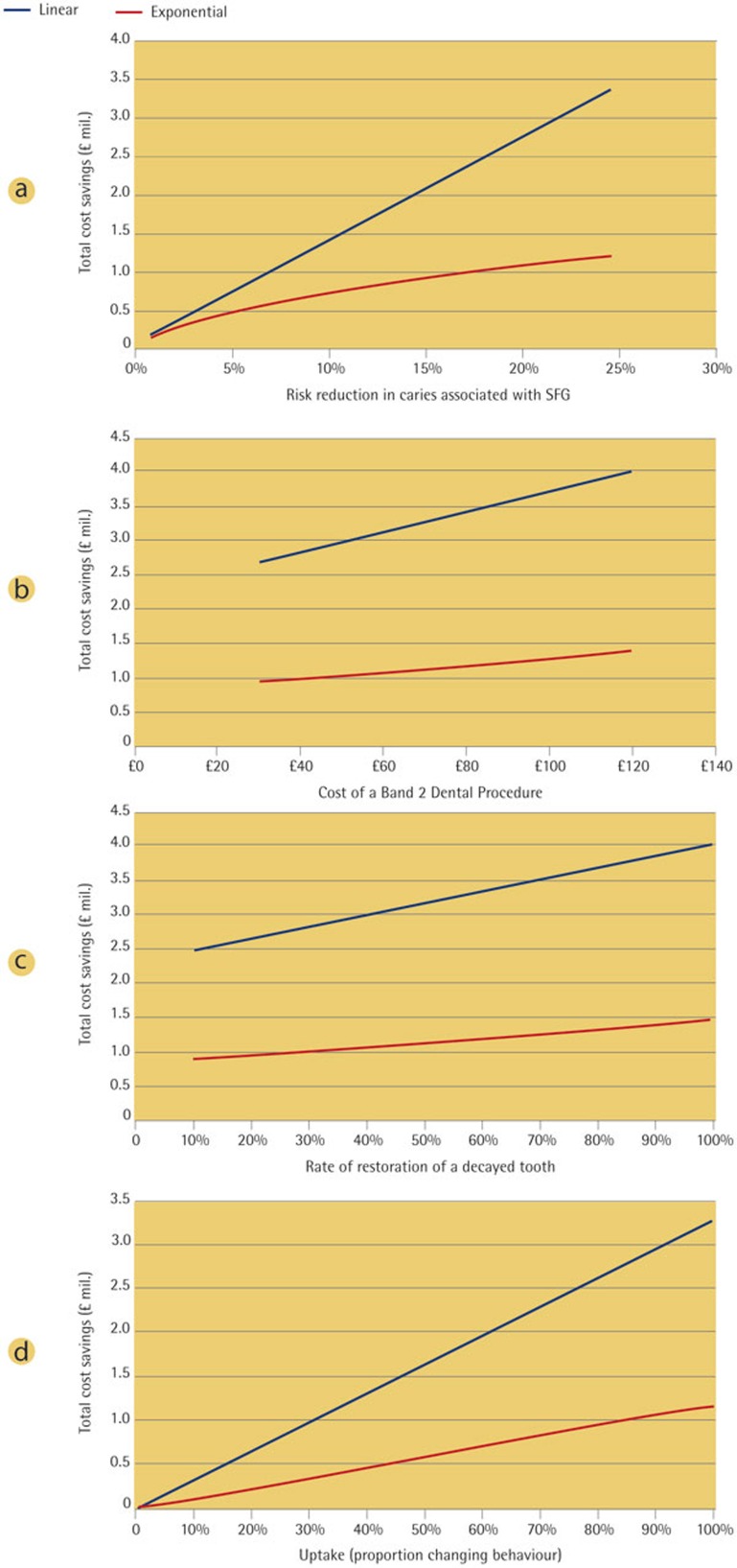
a) Sensitivity analysis – impact of the relative risk reduction in dental caries associated with the use of SFG on total cost savings. b) Sensitivity analysis – impact of the cost of a Band 2 dental procedure on total cost savings. c) Sensitivity analysis – impact of the restoration rate of decayed teeth on total cost savings. d) Sensitivity analysis – impact of SFG uptake on total cost savings

The final sensitivity analysis examined the effect of different levels of uptake of chewing SFG in the 12-year-old population. As expected, total cost savings in this population were shown to decrease as the proportion increasing their usage to twice per day (uptake) decreases. The potential cost savings were estimated to be between £0.6 and £1.6 million per year if 50% of the 12-year-old population increased their usage to twice per day. If the proportion increasing their usage was as low as 10%, then savings were estimated to be between £0.1 and £0.3 million per year (Fig. 2).

## Discussion

There is substantial literature showing that chewing SFG prevents the development of dental caries and this analysis shows that significant cost savings would be generated if usage of SFG were increased. If every member of the UK 12-year-old population increased their current SFG use by just one extra chewing occasion a day, potential cost savings of £1.1 million per year might be achieved. Therefore, a policy designed to encourage the use of SFG could lead to significant decreases in expenditure on dental care and result in a reduction in capacity pressure on the UK dental healthcare system.

Cost savings due to the use of SFG would be experienced by the NHS. However, the cost of the intervention would be borne either by the individual (their purchase of SFG) and the body taking responsibility for promoting SFG, (commercial or public health body). This has the result that SFG is always cost-saving to the NHS (assuming that it does not cause dental health to deteriorate, and that the NHS does not bear the cost of promoting SFG). The use of SFG incurs costs only to those individuals who benefit, at least in the short-term. Cost savings to those bearing the cost of the intervention would be achieved due to their avoiding dental treatment as adults. However, since most individuals use SFG for the non-dental benefits, such as the taste, to disguise bad breath, or to avoid hunger and not for the dental benefits, the dental benefits are a 'bonus' both to the individual and the NHS.

The analysis presented in this paper takes a 'whole population' approach (12-year-old population), where all members of a population are targeted, rather than those at-risk or with a particular need of oral disease prevention. With a national coverage campaign, SFG use is perhaps most likely to increase in those with current good oral health, as they are the group more likely to be health-conscious and receptive to health promotion messages. This group has less capacity to benefit, but health benefits would still be observed. The base models assume full compliance and uptake in each SFG scenario. In reality, this is unlikely. It is possible that uptake is likely to differ by level of deprivation. A sensitivity analysis of this parameter has demonstrated that cost savings would still be observed even with very low rates of uptake. School policies often prohibit the chewing of gum. The results of this study could support a review of such policies as the potential for SFG to offer substantial cost savings and health benefits and cost savings to the NHS is clear.

There are a number of limitations associated with this study. No UK clinical trials of SFG could be identified and therefore evidence was taken from a study based in Lithuania that was undertaken in 1994 to 1997. An epidemiological survey of children and adolescents in Lithuania in 1996 found that the mean DMFT among 12-year-olds was 4.9,^28^ a value substantially higher than the current mean DMFT of 12-year-olds in the UK. The average DMFT of children aged 12 in England is 0.7, and ranges from 1.0 in the most deprived quintile of the population to 0.5 in the least deprived quintile.^10^ It could be argued that similar reductions in dental caries are unlikely to be realised in a country with as low a prevalence of dental caries as the UK. Caries reductions achieved with SFG are likely proportional to the baseline prevalence *and* therefore the risk reduction will be a function of both baseline prevalence and chewing frequency. The sensitivity analysis undertaken as part of this study demonstrated that cost savings would not be as substantial as those estimated in the base case analysis, but would nevertheless still be generated even if only a 1% reduction in caries were achieved. Further research on the effects of SFG in a population with low levels of tooth decay, such as in the UK, would be beneficial for estimating both the clinical and economic impact of chewing SFG.

Evidence for the effects of chewing SFG at different frequencies was also not available, so two frequency-response models were developed. These scenarios reflect that there are many variables in caries progression (only one of which is the use of SFG), which are not always linear. In both scenarios, it was conservatively assumed that the caries risk reduction presented in the study is the maximum benefit that can be achieved regardless of the quantity and frequency of chewing. This effect is demonstrated in the studies undertaken by Mäkinen, which showed no significant difference between chewing three and five times per day.^16^,^17^ The linear response relationship represents a more optimistic scenario compared to the exponential scenario, in which SFG must be chewed more often before the benefits on dental caries are realised. There is little evidence as to whether the relationship between dose and effect is linear or exponential. It is possible that it may change from exponential to linear as the baseline decay rate increases, that is, the dose response is more noticeable in high caries prevalence populations.

The focus of this study was to capture the potential short-term cost savings to the NHS, specifically in a hypothetical population of 12-year-olds. While they are very important, the short- and long-term health effects of the intervention were not analysed. We made no attempt to include any measure of the disutility associated with dental caries, so our results are limited to a description of the potential cost savings. It is important to remember that the effects of tooth decay and tooth loss can be severe, so in addition to saving valuable dental health care resources, chewing SFG also improves the dental health of the nation and enhances well-being and oral health related quality of life. Furthermore, improving the dental health in young people is likely to have extremely long term effects as young people with poor oral health are likely to have higher levels of tooth decay throughout their adult life.

The model attempts to capture the potential cost savings that may occur due to the prevention of caries development over a relatively short time horizon, and in a small subset of the population. Adopting a longer-term time horizon for the study would have allowed for the benefits of the intervention to be more fully captured, because the treatment of a decayed tooth is rarely limited to the initial restoration. Evidence suggests that restorations have a limited life span, and that once a tooth is restored, the filling is likely to be replaced many times in the patient's lifetime.

Our analysis is therefore conservative in its approach, and the potential cost savings over a life time, as well as the long term health benefits, have been consciously underestimated. It is therefore likely that, by increasing use of SFG and thereby reducing the level of caries development, even greater cost savings in the long-term will be realised than those estimated in this analysis.

This study shows that if levels of SFG usage in the teenage population in the UK could be increased, substantial cost savings might be achieved.

## Figures and Tables

**Table 1:** Chewing frequency behaviours in the UK in children aged 10 to 14 (2014)

Group	Group definition	Annual number of chewing occasions	Proportion of SFG users
Group 1: No use	No use of SFG	0	6%
Group 2: Infrequent use	Less than one chewing occasion per week	26	22%
Group 3: Light use	Between 1 and 4 chewing occasions per week	130	36%
Group 4: Moderate use	Between 5 and 10 chewing occasions per week	390	22%
Group 5: Frequent use	More than 10 chewing occasions per week	780	14%

**Table 2:** Baseline risk of decay in 12-year-olds

Parameter	Value
Children examined	89,442
Average of DMFT	0.7
Number with caries experience (DMFT >0)	30,181
Proportion with caries experience (%DMFT >0)	33.74%
With caries experience, number with extraction experience (MT >0)	3,165
With caries experience, proportion with extraction experience (%MT >0)	10.49%
With caries experience, number with fillings present (FT >0)	18,158
With caries experience, proportion with fillings present (%FT >0)	20.30%

**Table 3:** Clinical studies on SFG

Study	Country of study	N	Population	Intervention	Follow-up period	Baseline caries	Control	Reduction in caries (%)
Möller 1973	Denmark	340	School children	Sorbitol gum	2 years	NR	No gum	10%
Scheinin 1975	Finland	100	Young adults	Xylitol gum	1 year	NR	Sucrose gum	91%
Glass 1983	USA	540	Children aged 7–11	Sorbitol gum twice a day	2 years	NR	No gum	2%
Isokangas 1989	Finland	324	Children aged 11–12	Xylitol gum	5 years	NR	No gum	45%
Kandelman 1990	Canada	274	Children aged 8–9	15% and 65% Xylitol gum	2 years	NR	No gum	61 – 66%
Mäkinen 1995	Belize	1,277	Children aged 10	Sorbitol, xylitol or combinations	40 months	NR	No gum	17 – 71%
Mäkinen 1996	Belize	510	Children aged 6	Sorbitol, xylitol or combinations	24 months	NR	No gum	28 – 69%
Beiswanger 1998	Puerto Rico	1,402	Children in grades 5–7	Sorbitol gum, daily after meals.	2 years	NR	No gum	12%
Alanen 2000	Estonia	740	Children aged 10	Xylitol gum	3 years	Control group DMFS: 2.18 (SD 3.30)	No gum	54%
						Xylitol group DMFS: 2.10 (SD 2.55)		
						Measurement excludes surfaces with incipient caries		
Szöke 2001	Hungary	547	School children aged 8–13	Sorbitol stick, daily after meals	2 years	Control group DMFS: 1.94 (2.85)	No gum	33%
						Gum group DMFS: 1.69 (SD 2.64)		
						Measurement excludes surfaces with incipient caries		
Machiulskiene 2001	Lithuania	432	Children aged 9–14	Sorbitol, xylitol, HIS gum	3 years	Control group DMFS: 6.4 (SD 4.3)	No gum	25 – 33%
						Xylitol gum group DMFS: 5.0 (SD 3.9)		
						Measurement includes all stages of caries formation		
Kovari 2003	Finland	921	Children in day care centres	Xylitol gum	6 years	NR	No gum	Paper could not be retrieved
Peng 2004	China	1,143	Children aged 6–7	Sorbitol, xylitol, carbamide gum	2 years	Control group DMFS: 0.05 (SD 0.30)	No gum	42%
						Gum group DMFS: 0.07 (SD 0.32)		
						Measurement includes all stages of caries formation		
Morgan 2008	Australia	2,720	Children aged 11–13	CPP-ACP gum	2 years	Control group D1MFS: 2.80 (SD 3.85)	SFG	17%
						Gum group D1MFS: 2.76 (SD 3.79)		
						Measurement includes all stages of caries formation		
Tao 2013	China	157	Children aged 8–9	Tea polyphenol gum	2 years	Control group DMFS: 0.36 (SD 0.79)	No gum	58%
						Gum group DMFS: 0.56 (SD 1.13)		
						Measurement includes all stages of caries formation		

**Table 4a:** Results – Scenario 1

	Base case	Hypothetical scenario (linear model)	Hypothetical scenario (exponential model)
Extraction costs	£22,948,628	£20,702,245	£22,148,906
Restoration costs	£10,427,070	£9,406,391	£10,063,704
Total costs	£33,375,698	£30,108,637	£32,212,610
Total savings for the population		£3,267,062	£1,163,089
Average savings per person		£4.77	£1.70

**Table 4b:** Results – Scenario 2

	Base case	Hypothetical scenario (linear model)	Hypothetical scenario (exponential model)
Extraction costs	£22,948,628	£21,018,002	£22,234,639
Restoration costs	£10,427,070	£9,549,860	£10,102,659
Total costs	£33,375,698	£30,567,863	£32,337,298
Total savings for the population		£2,807,836	£1,038,400
Average savings per person		£4.10	£1.52

**Table 4c:** Results – Scenario 3

	Base case	Hypothetical scenario (linear model)	Hypothetical scenario (exponential model)
Extraction costs	£22,948,628	£17,502,923	£17,313,302
Restoration costs	£10,427,070	£7,952,729	£7,866,571
Total costs	£33,375,698	£25,455,652	£25,179,873
Total savings for the population		£7,920,046	£8,195,826
Average savings per person		£11.57	£11.97

## Notes

**Refereed Paper**

## References

[b1] Jensen M E. Effects of chewing sorbitol gum and paraffin on human interproximal plaque pH. Caries Res 1986; 20: 503–509. 3465444 10.1159/000260981

[b2] Manning R H, Edgar W M. pH changes in plaque after eating snacks and meals, and their modification by chewing sugared-or sugarfree gum. Br Dent J 1993; 174: 241–244. 8461202 10.1038/sj.bdj.4808141

[b3] Llena-Puy C. The role of saliva in maintaining oral health and as an aid to diagnosis. Med Oral Patol Oral Cir Bucal 2006; 11: E449–E455. 16878065

[b4] Stookey G K. The effect of saliva on dental caries. J Am Dent Assoc 2008; 139 (Suppl): 11S–17S. 18595200 10.14219/jada.archive.2008.0347

[b5] Mickenautsch S, Leal S C, Yengopal V, Bezerra A C, Cruvinal V. Sugar-free chewing gum and dental caries: A systematic review. J Appl Oral Sci 2007; 15: 83–88. 19089107 10.1590/S1678-77572007000200002 PMC4327235

[b6] EFSA Panel on Dietetic Products, Nutrition and Allergies (NDA). Scientific Opinion on the substantiation of a health claim related to sugar-free chewing gum and reduction of tooth demineralisation which reduces the risk of dental caries pursuant to Article 14 of Regulation (EC) No 1924/20061. EFSA J 2010; 8: 1775. Available online http://www.efsa.europa.eu (accessed January 2016).

[b7] NHS England. 2014. Improving dental care – a call to action. 2014. Available online at http://www.england.nhs.uk/wp-content/uploads/2014/02/imp-dent-care.pdf (accessed January 2016).

[b8] Prescribing and Primary Care, Health and Social Care Information Centre. NHS dental statistics for England: 2013/2014. 2014. Available online at http://www.hscic.gov.uk/catalogue/PUB14738/nhs-dent-stat-eng-13-14-rep.pdf (accessed January 2016).

[b9] Listl S, Galloway J, Mossey P A, Marcenes W. Global economic impact of dental diseases. J Dent Res 2015; 94: 1355–1361. 26318590 10.1177/0022034515602879

[b10] Public Health England. The NHS dental epidemiology programme for England: oral health survey of 12 year old children 2008/2009. 2010. Available online at http://www.nwph.net/dentalhealth/reports/Report_NHS_DEP_for_England_OH_Survey_12yr_2008-09.pdf (accessed January 2016).

[b11] Moller I J, Poulsen S. The effect of sorbitol-containing chewing gum on the incidence of dental caries; plaque and gingivitis in Danish schoolchildren. Community Dent Oral Epidemiol 1973; 1: 58–67. 4153719 10.1111/j.1600-0528.1973.tb01861.x

[b12] Scheinin A, Makinen K K, Tammisalo E, Rekola M. Turku sugar studies XVIII. Incidence of dental caries in relation to 1-year consumption of xylitol chewing gum. Acta Odontol Scand 1975; 33: 269–278. 1067728 10.3109/00016357509004632

[b13] Glass R L. A two-year clinical trial of sorbitol chewing gum. Caries Res 1983; 17: 365–368. 6347385 10.1159/000260689

[b14] Isokangas P, Tiekso J, Alanen P, Makinen K K. Long-term effect of xylitol chewing gum on dental caries. Community Dent Oral Epidemiol 1989; 17: 200–203. 2758793 10.1111/j.1600-0528.1989.tb00611.x

[b15] Kandelman D, Gagnon G. A 24-month clinical study of the incidence and progression of dental caries in relation to consumption of chewing gum containing xylitol in school preventive programs. J Dent Res 1990; 69: 1771–1775. 2229617 10.1177/00220345900690111201

[b16] Makinen K K, Makinen P L, Pape H R . Stabilisation of rampant caries: polyol gums and arrest of dentine caries in two long-term cohort studies in young subjects. Int Dent J 1995; 45(Suppl 1): 93–107. 7607749

[b17] Makinen K K, Hujoel P P, Bennett C A, Isotupa K P, Makinen P L, Allen P. Polyol chewing gums and caries rates in primary dentition: a 24-month cohort study. Caries Res 1996; 30: 408.17. 8946097 10.1159/000262352

[b18] Beiswanger B B, Boneta A E, Mau M S, Katz B P, Proskin H M, Stookey G K. The effect of chewing SFG after meals on clinical caries incidence. J Am Dent Assoc 1998; 129: 1623–1626. 9818584 10.14219/jada.archive.1998.0113

[b19] Alanen P, Isokangas P, Gutmann K. Xylitol candies in caries prevention: results of a field study in Estonian children. Community Dent Oral Epidemiol 2000; 28: 218–234. 10830649 10.1034/j.1600-0528.2000.280308.x

[b20] Szöke J, Bánóczy J. Effect of after-meal sucrose-free gum-chewing on clinical caries. SADJ 2001; 80: 1725–1729. 10.1177/00220345010800080901 11669483

[b21] Machiulskiene V, Nyvad B, Baelum V. Caries preventive effect of sugar-substituted chewing gum. Community Dent Oral Epidemiol 2001; 29: 278–288. 11515642 10.1034/j.1600-0528.2001.290407.x

[b22] Kovari H, Pienihäkkinen K, Alanen P. Use of xylitol chewing gum in daycare centers: a follow-up study in Savonlinna, Finland. Acta Odontol Scand 2003; 61: 367–370. 14960009 10.1080/00016350310007806

[b23] Peng B, Petersen P E, Bian Z, Tai B, Jiang H. Can school-based oral health education and a sugar-free chewing gum program improve oral health? Results from a two-year study in PR China. Acta Odontol Scand 2004; 62: 328–332. 15848976 10.1080/00016350410010036

[b24] Morgan M V, Adams G G, Bailey D L, Tsao C E, Fischman S L, Reynolds E C. The anticariogenic effect of SFG containing CPP-ACP nanocomplexes on approximal caries determined using digital bitewing radiography. Caries Res 2008; 42: 171–184. 18446025 10.1159/000128561

[b25] Tao D Y, Shu C B, Lo E C, Lu H X, Feng X P. A randomized trial on the inhibitory effect of chewing gum containing tea polyphenol on caries. J Clin Pediatr Dent 2013; 38: 67–70. 24579286 10.17796/jcpd.38.1.c0tm02w572488064

[b26] Curtis L. Unit costs of health and social care. Canterbury: Personal Social Services Research Unit, University of Kent, 2014.

[b27] Office for National Statistics. Population estimates by age and sex, mid-2014. 2015. Available online at http://www.ons.gov.uk/ons/taxonomy/index.html?nscl=Population+Estimates+by+Age+and+Sex (accessed January 2016).

[b28] Aleksejuniene J, Arneberg P, Eriksen H M. Caries prevalence and oral hygiene in Lithuanian children and adolescents. Acta Odontol Scand 1996; 54: 75–80. 8669245 10.3109/00016359609003513

